# Low Expression of Selenoprotein S Modulates Osteogenic Differentiation Through Bidirectional Regulation of the *SP7*–*HSP47*/*COL1A1*/*SPARC* Axis

**DOI:** 10.3390/cimb47090677

**Published:** 2025-08-23

**Authors:** Hao Wu, Yun-Shan Zhao, Chun-Shen Li, Jing-Yi Shi, Yi Li, Liang-Qiu-Yue Zhong, Yan Liu, Xi Chen

**Affiliations:** Department of Stomatology, First Affiliated Hospital, College of Medicine, Xi’an Jiaotong University, 277 Yanta West Road, Xi’an 710061, China; wuhaowuh@stu.xjtu.edu.cn (H.W.);

**Keywords:** selenoprotein S, osteogenic differentiation, BMSCs, collagen synthesis

## Abstract

Previous studies revealed that low expression of Selenoprotein S (SELS) could enhance osteogenic differentiation, but the underlying mechanisms remain unclear. In this study, we aimed to elucidate the role of SELS and its transcription-factor-based regulatory mechanism during osteogenic differentiation. In comparison with 12-week-old mice, which represent the stage of stable osteogenic differentiation, 3-week-old mice, representing the active ossification stage, showed significantly higher levels of SELS in the mandible. Transcriptomic analysis revealed that SELS is primarily associated with extracellular matrix organization and collagen biosynthesis during mandibular development. In bone marrow mesenchymal stem cells (BMSCs) with SELS knockdown, *SP7* levels were elevated after 7 days of osteogenic induction in vitro. Consistently, immunohistochemical and immunofluorescence staining confirmed increased *SP7* expression in the mandibles of 7-week-old *Sels* knockout mice. Dual-luciferase reporter assays and chromatin immunoprecipitation (ChIP) analysis demonstrated that *SP7* directly binds to the heat shock protein 47 (*HSP47*) promoter and negatively regulates its transcription. Consequently, upregulation of *SP7* following SELS knockdown led to downregulation of *HSP47* and concurrent upregulation of the *SP7* downstream targets, collagen type I alpha 1 chain (*COL1A1*) and Secreted protein acidic and rich in cysteine (*SPARC*). SELS expression is upregulated during active osteogenesis. Low expression of SELS regulates osteogenic differentiation in a bidirectional and fine-tuned manner through the *SP7*–*HSP47*/*COL1A1*/*SPARC* axis.

## 1. Introduction

Selenium is a vital trace element required for maintaining human health, primarily exerting its effects through its involvement in the synthesis of selenoproteins. These proteins play critical roles in antioxidant defense and endocrine regulation, with alterations in their expression impacting the functions of various cell types. Notably, multiple selenoproteins exhibit functions tightly linked to collagen synthesis and osteogenic differentiation, underscoring their crucial roles in bone tissue formation and regeneration. Among the 25 selenoproteins identified in humans, SELS predominantly resides in the endoplasmic reticulum, near the nucleus, and in the Golgi apparatus [1]. It is implicated in inflammation, protein quality control, and cellular stress management [2]. Furthermore, SELS participates in endoplasmic-reticulum-associated degradation (ERAD), facilitating the transfer of unfolded or misfolded proteins to the cytoplasm for proteasomal degradation [3]. Beyond these roles, its regulation of transcription factors and cytokines is also crucial for maintaining cellular functions [4]. In addition, it has been reported that silencing SELS expression leads to the expression of Runx2, ALP, and COL1 in vascular smooth muscle cells, implying a potential role of SELS in osteogenic differentiation [5].

Bone metabolism is a critical physiological function, with the long-term balance between bone resorption and bone formation being essential for overall health [6]. In the process of bone formation, osteoblasts secrete an extracellular matrix predominantly composed of type I collagen fibers, which serves as a structural scaffold for subsequent matrix mineralization [7]. Consequently, collagen synthesis is essential for the formation and maturation of bone. Importantly, bone formation is orchestrated by a complex and tightly regulated network of signaling molecules, including various cytokines and transcription factors, which coordinate the differentiation and functional activity of osteogenic cells. This equilibrium is largely maintained through the stable expression of osteogenesis-related transcription factors and cytokines. *SP7*, an osteoblast-specific transcription factor, contains a zinc finger domain and plays a pivotal role in osteogenic differentiation [8]. It is expressed in both osteoblasts and chondrocytes and is often used as a marker of osteogenic differentiation due to its strong correlation with this process [9]. Functioning as a transcription factor, *SP7* influences the expression of several molecules involved in osteogenic differentiation and related functions. Its downstream target genes include collagen type I alpha 1 chain (*COL1A1*), secreted protein acidic and rich in cysteine (*SPARC*), osteocalcin (OCN), and bone sialoprotein (BSP) [10,11,12], all closely associated with collagen synthesis, osteoblast differentiation, and cellular mineralization. Beyond its positive regulation of these target molecules, *SP7* also exerts a reverse regulatory effect on certain molecules. Specifically, reduced *SP7* expression leads to increased expression of fibrillin-2 and periostin [13]. The diverse regulatory functions of *SP7* on downstream molecules highlight its functional versatility and suggest that the underlying regulatory mechanisms of *SP7* remain incompletely understood.

Our previous study revealed a discrepancy between the mRNA and protein expression levels of SELS during osteogenic induction and showed that low expression of SELS facilitates osteogenic differentiation [14]. However, the precise molecular mechanisms involved remain to be elucidated. In this study, we aimed to investigate the expression pattern of SELS at different stages of osteogenic differentiation and explore the effects of low SELS expression on the osteogenic differentiation of BMSCs in vitro, as well as the mechanisms of these effects, and its impact on mandibular osteogenesis in vivo.

## 2. Materials and Methods

### 2.1. Cell Culture

Bone Marrow Mesenchymal Stromal Cells (BMSCs, Procell, Wuhan, China) were maintained in α-MEM (Gibco, Grand Island, NE, USA) formulated with 10% fetal bovine serum (FBS; Cyagen, Guangzhou, China) and 1% penicillin–streptomycin (Beyotime, Shanghai, China), while 293T cells (Procell, Wuhan, China) were grown in DMEM (Gibco, Grand Island, NE, USA) under the same conditions, and the medium was renewed every two days. The cell culture incubator was kept at 37° and 5% CO_2_. When the cultures reached 90% confluency, the cells were passaged in a ratio of 1:4.

### 2.2. Cell Transfection

After subculturing and plating the cells, lentiviral solution and the transfection reagent polybrene (Beyotime, Shanghai, China) were added to the cultures when the cell density reached approximately 30%. The cells were co-cultured for about 20 h, after which the medium was changed and the cells were maintained for a further 24 h. When the cell confluency was approximately 90%, the cells were subcultured again. Once the cells adhered, puromycin (Beyotime, Shanghai, China) was added, yielding a selection concentration of 5 µg/mL, and the selected cells were used for subsequent experiments. In this study, the lentiviral knockdown group was designated as “sh-Sels,” while the blank control group was designated as “sh-NC.” The SELS interference sequence used in this study was CAGGAAGATCTAAATGCCCAA.

### 2.3. Osteogenic Induction

Osteogenic induction medium was prepared by supplementing the complete culture medium with dexamethasone, β-glycerophosphate, and ascorbic acid (Beyotime, Shanghai, China), and the final concentrations of the three components in the induction medium were adjusted to 0.1 μmol/L, 10 mmol/L, and 50 μg/mL, respectively. During the osteogenic induction process, the induction medium was replaced every other day.

### 2.4. Quantitative Real-Time PCR

Total RNA was purified using a fast RNA isolation kit (Fastgen, Shanghai, China) and promptly converted into cDNA via reverse transcription. Gene expression levels were quantified via the 2^−ΔΔCT^ approach. The primer sequences applied in this study are shown in Table 1.

### 2.5. Animals

*Sels* knockout mice, obtained from Cyagen (Guangzhou, China), were generated via the CRISPR/Cas9-mediated editing of the *Selenos* gene. Based on genotyping results, homozygous *Sels* knockout mice and wild-type controls (*n* = 5 per group, regardless of sex) were allocated to the corresponding experimental groups, designated as “WT” and “*Sels*^−/−^”. The mice were maintained under specific pathogen-free conditions and provided with food ad libitum, with 6–8 mice per cage. The ambient temperature was set to 23 °C, and a 12 h light/dark cycle was automatically maintained. Mice were euthanized via cervical dislocation at 7 weeks of age, and mandibles were carefully harvested. Soft tissues were thoroughly removed as much as possible during dissection, while ensuring the integrity of the dentition. Moreover, 3- and 12-week-old wild-type C57BL/6 mice (*n* = 5 per group, regardless of sex) were purchased from the Experimental Animal Center of Xi’an Jiaotong University, designated as “3W” and “12W”.

### 2.6. Western Blotting

Protein extraction was performed using RIPA buffer (Yamei, Shanghai, China), denatured via boiling, and separated via electrophoresis on a polyacrylamide gel (PAGE, Yamei, Shanghai, China). The mandibular bone tissue was ground into powder using cryogenic high-speed grinding, followed by routine protein extraction. After transfer to a PVDF membrane (Merck Millipore, Billerica, MA, USA), the proteins were blocked in 10% skim milk and subjected to incubation with primary and secondary antibodies. Finally, the membrane was immersed in ECL luminescent solution (MedChem Express, Monmouth Junction, NJ, USA) and imaged using a gel imaging system. The primary antibodies employed for the experiments included the following: anti-SELS (1:1000, Proteintech, Wuhan, China), anti-*SP7* (1:1000, Immunoway, Suzhou, China), anti-*HSP47*(1:1000, Immunoway, Suzhou, China), anti-*SPARC* (1:1000, Immunoway, Suzhou, China), anti-FLAG (1:5000, Immunoway, Suzhou, China), and anti-β-ACTIN (1:10,000, Abclonal, Wuhan, China).

### 2.7. Immunohistochemical Staining

The mandibles were fixed in 4% formalin (Service, Wuhan, China) at room temperature for one week. Subsequently, the mandibles were decalcified by immersion in 13% ethylenediaminetetraacetic acid (EDTA) solution (Service, Wuhan, China) at a volume at least 10 times greater than that of the tissue, using a shaker at room temperature, with the solution replaced every 2 days for a total of 14 days. Following decalcification, the tissues were embedded in paraffin. Sagittal sections approximately 5 μm thick were prepared along the dental arch to ensure the inclusion of the first and second molars. Sections were dewaxed sequentially in xylene and graded ethanol series, before being subjected to antigen retrieval via incubation in compound repair solution (Boster, Wuhan, China) at 37 °C for 30 min. After two washes with PBS (5 min each), endogenous peroxidase activity was blocked via incubation with 3% hydrogen peroxide (Boster, Wuhan, China) at 37 °C for 30 min, followed by two additional PBS washes (5 min each). Subsequently, sections were blocked with goat serum (Boster, Wuhan, China) at 37 °C for 30 min. After briefly draining the serum, the sections were incubated with the primary antibody for 2 h at 37 °C. The sections were treated with secondary antibodies, proceeding with DAB staining (ZSBio, Beijing, China) for 1 h. Later, the sections were stained with hematoxylin, subjected to routine dehydration, and mounted with neutral gum. The primary antibody utilized was anti-*SP7* (1:100; Immunoway, Suzhou, China). The furcation area of the first molar was selected as the region of interest (ROI) to assess and analyze osteogenic differentiation within the mandible.

### 2.8. Immunofluorescence

The pretreatment of tissue sections followed the same procedure as that used for immunohistochemistry. Following hydration, the sections were incubated with 0.1% Triton X-100 (Beyotime, Shanghai, China) for 15 min, followed by antigen retrieval at 37 °C for 30 min and blocking at 37 °C for 30 min. The sections were placed with the primary antibody and left to incubate at 37 °C for 2 h. Later, sections were kept with a fluorescence-conjugated secondary antibody at room temperature for 1 h, followed by nuclear counterstaining with DAPI (Beyotime, Shanghai, China) at room temperature for 10 min. Coverslips were then mounted with an anti-fade mounting medium (Beyotime, Shanghai, China). The primary antibodies applied were anti-*SP7* (1:100; Immunoway, Suzhou, China) and *COL1A1* (1:100; Immunoway, Suzhou, China). The furcation area of the first molar was selected as the region of interest (ROI) to assess and analyze osteogenic differentiation within the mandible.

### 2.9. Transcription Factor Target Gene Screening and Binding Sequence Prediction

To investigate the molecular mechanisms via which *SP7* regulates osteogenic differentiation, we employed bioinformatics approaches to predict potential target genes of *SP7*. We utilized the GTRD [15] and Cistrome DB [16] databases to predict potential target genes of *SP7*. By cross-referencing these predictions with the gene list from the osteogenesis-related PCR array, we identified *HSP47* as a potential target molecule. Furthermore, the JASPAR database [17] was utilized to identify potential binding sites of *SP7* on the *HSP47* promoter, ultimately identifying five potential binding sites for experimental verification. Candidate *SP7*-binding regions in the *HSP47* promoter are listed in Table 2.

### 2.10. The Dual-Luciferase Reporter Assay

Based on bioinformatics predictions, we performed dual-luciferase reporter assays to examine the potential interaction between *SP7* and *HSP47*. The plasmids used in this study included *SP7* overexpression plasmids (oe), containing the wild-type promoter sequence (wt), a mutant binding site sequence (mut), and a Renilla luciferase plasmid (re). A seeding density corresponding to 30% confluency was used in 24-well plates, followed by a 24 h incubation. To initiate transfection, 3 μL of Lipo8000 (Beyotime, Shanghai, China) was mixed with 50 μL of Opti-MEM and kept undisturbed for 5 min, followed by the addition of a pre-mixed solution containing three plasmids: 450 ng of oe, 450 ng of wt or mut, and 100 ng of re. The solution was mixed thoroughly and kept at ambient temperature for 20 min. The resulting mixture was applied to the pre-seeded cells and kept for 24 h, with 1000 ng of plasmid DNA per well. After an additional 24 h of incubation, luciferase activity was assessed following cell lysis using a luciferase assay kit (Vazyme, Nanjing, China). The firefly luciferase luminescent signal for each sample was standardized against the Renilla luciferase signal. This experiment was performed in vitro using 293T cells.

### 2.11. Chromatin Immunoprecipitation

To determine whether there is a direct regulatory relationship between *SP7* and *HSP47*, ChIP assays were performed in BMSCs as an in vitro experiment. ChIP were conducted with a ChIP Assay Kit (Beyotime, Shanghai, China). An anti-Flag antibody (1 μg; Immunoway, Suzhou, China) was used for immunoprecipitation, and normal mouse immunoglobulin G (IgG, 1 μg, Proteintech, Wuhan, China) served as the negative control (NC). PCR amplification was carried out according to the PCR mix instructions (Abclonal, Wuhan, China). The primer sequences used were as follows: Forward: 5′-GACCCCTGCCTCTGACTTTTT-3′; Reverse: 5′-CCCCAGTAGATCCATTGCCTA-3′.

### 2.12. Transcriptome Analyses

The transcriptomic analysis was based on both publicly available data derived from the GEO database (GSE121197, accessed on 1 February 2025) and a reanalysis of transcriptome sequencing data generated by our research group [14]. The GSE121197 dataset consists of transcriptome sequencing data from tissues of Tgfbr2 mutant and control embryos at stage E12.5. From the GSE121197 dataset, we selected mandibular bone transcriptome data from the control group for reanalysis. Samples were grouped based on their mandibular sampling sites. Six samples from two groups named “Mouse E12.5 Control Tgfbr2 Mandible Proximal” and “Mouse E12.5 Control Tgfbr2 Mandible Distal” were selected for further analysis. This part of the study was conducted in vivo. After performing differential gene expression analysis, we further divided the samples into high- and low-SELS-expression groups based on the median expression level of SELS. Subsequent analyses included differential gene expression analysis and Gene Ontology (GO) and Kyoto Encyclopedia of Genes and Genomes (KEGG) enrichment analyses of the identified DEGs. Bioinformatics analysis was conducted using R software (version 4.3.2). GO and KEGG pathway analyses were conducted using the clusterProfiler package, and the ggplot2 package was used to create visualizations, including creating volcano plots, Venn diagrams, and bubble charts. DEGs were identified using a threshold of *p* < 0.05. DEGs from a previous transcriptome analysis were intersected with an osteogenesis-related PCR array gene list to identify osteogenic DEGs. GO and KEGG analyses were then performed to determine their functions. This study was conducted in vitro.

### 2.13. Statistical Analysis

Statistical differences were assessed using an unpaired two-tailed Student t-test, assuming data normality and applying a 95% confidence interval. The results of the luciferase reporter assay were statistically analyzed using one-way ANOVA. The analysis was conducted in GraphPad Prism software (version 6.0.0). Values are reported as the mean ± standard deviation (SD). 

## 3. Results

### 3.1. Expression of SELS During the Active Phase of Osteogenesis

**Figure 1:**
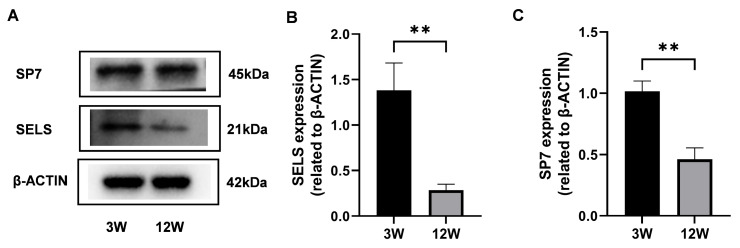
Expression of SELS at different stages of osteogenic differentiation. (**A**) Protein levels of SELS and *SP7* in mandibular bone tissues from 3-week-old and 12-week-old mice were assessed via Western blotting. (**B**,**C**) Densitometric analysis of SELS (**B**) and *SP7* (**C**) expression normalized to β-ACTIN. Data are represented as means ± SD. (** *p* < 0.01).

To explore the expression dynamics of SELS during osteogenesis, proteins were extracted from the mandibular bones of 3-week-old and 12-week-old mice, representing the active and stable phases of osteogenesis, respectively. Western blot analysis revealed that *SP7* expression was significantly higher in 3-week-old mice compared with 12-week-old mice (Figure 1A,C), confirming increased osteogenic activity at this stage. Based on this validation, we further examined SELS expression and found that it was also significantly elevated in 3-week-old mice (Figure 1A,B), suggesting a close association between SELS expression and the active phase of osteogenic differentiation.

### 3.2. SELS Expression Patterns and Related Transcriptomic Changes During Mandibular Osteogenesis

**Figure 2:**
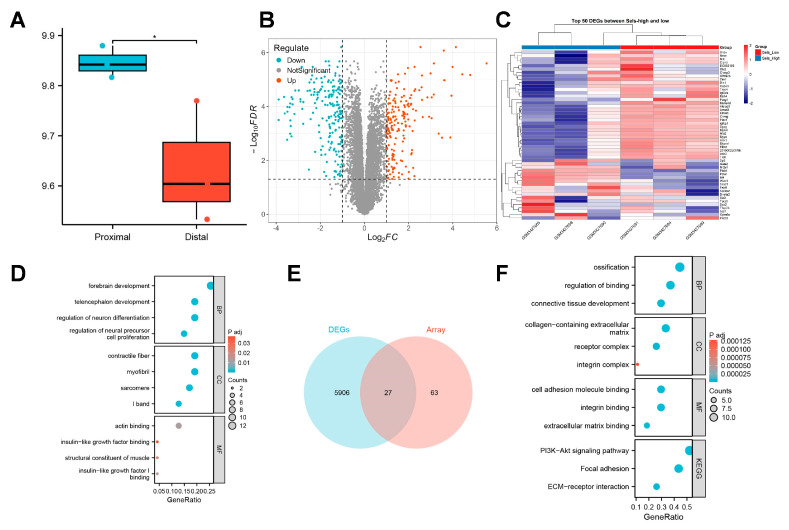
Transcriptomic analysis of SELS-related osteogenic regulation. (**A**) The expression levels of SELS in different regions of the mandible during osteogenic differentiation. Blue bars represent proximal mandibular tissues, whereas red bars indicate distal mandibular tissues. (**B**) A volcano plot showing the distribution of DEGs between high- and low-SELS-expression groups. (**C**) A heatmap displaying the expression patterns of the top 50 DEGs. (**D**) GO enrichment analysis of DEGs. (**E**) Differentially expressed osteogenesis-related genes identified in the transcriptome dataset of BMSCs with low SELS expression. (**F**) GO and KEGG enrichment analyses of the osteogenesis-related DEGs in BMSCs with low SELS expression. GO: Gene Ontology; BP: Biological Process; CC: Cellular Component; MF: Molecular Function; KEGG: Kyoto Encyclopedia of Genes and Genomes. Data are represented as means ± SD, *n* = 3. (* *p* < 0.05).

To elucidate the regulatory mechanisms through which SELS influences osteogenic differentiation, we conducted a preliminary analysis using publicly available transcriptomic data. This dataset collected sequencing data from mandibular tissues of 12.5-day mouse embryos. In this study, sequencing data from the proximal and distal parts of the mandible in the control group were included, with three samples per group, totaling six samples, labeled as “proximal” and “distal,”, respectively. Given that different regions of the mandible at various developmental stages represent distinct levels of osteogenic differentiation, we first assessed the expression pattern of SELS during mandibular development. A significant difference in SELS expression was observed between the proximal and distal regions of the mandible (Figure 2A). Based on the median expression level of SELS, samples were divided into high- and low-expression groups for further analysis. DEGs were visualized using a volcano plot (Figure 2B), and a heatmap of the top 50 DEGs showed clear clustering between the groups (Figure 2C). GO analysis revealed enrichment in pathways related to neural tissue formation and insulin-like growth factor (IGF) signaling (Figure 2D). In addition, we reanalyzed a previously published transcriptomic dataset from our research group. Differential expression analysis was performed, and 27 osteogenesis-related DEGs were identified via intersection with a curated osteogenic gene set (Figure 2E). Subsequent GO and KEGG enrichment analyses revealed that these genes were predominantly associated with extracellular matrix organization and cellular mineralization, consistent with the findings from the public dataset (Figure 2F).

### 3.3. Histological Analysis of the Impact of Low SELS Expression on Mandibular Bone

**Figure 3:**
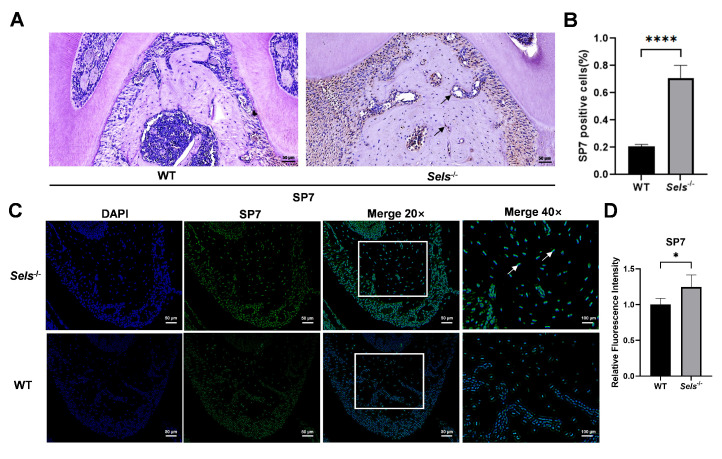
Histological analysis of the effects of *Sels* knockout on the mandibular bone. (**A**) Immunohistochemical staining of *SP7* in the furcation region of the mandibular bone from *Sels* knockout (experimental) and wild-type (control) mice. Representative positive cells are indicated by arrows. (**B**) Quantification of *SP7*-positive cells presented as the percentage of positive cells in each group. (**C**) Immunofluorescence staining of *SP7* in the furcation region of the mandibular bone from *Sels* knockout (experimental) and wild-type (control) mice. Images within the boxed areas show low-magnification views, with corresponding high-magnification images displayed at the indicated locations. Typical positive cells are marked with arrows. (**D**) Quantitative analysis of relative *SP7* fluorescence intensity. “WT” and “*Sels*^−/−^”: wild-type and *Sels* knockout mice. Data are represented as means ± SD (*n* = 5), scale bars: 50 and 100 μm. (* *p* < 0.05, **** *p* < 0.0001).

To explore the role of *Sels* knockout in mandibular osteogenic differentiation, 7-week-old *Sels* knockout mice (experimental group) and wild-type mice (control group) were analyzed. The furcation region of the mandibular first molar was selected as the ROI. Immunohistochemical staining revealed a significantly higher percentage of *SP7*-positive cells in the *Sels* knockout group compared with wild-type controls (Figure 3A,B). Consistently, immunofluorescence staining showed increased *SP7* fluorescence intensity in the knockout group (Figure 3C,D). In the immunofluorescence images, *SP7* signals overlapped with DAPI, confirming its nuclear localization. These results demonstrate that loss of SELS enhances *SP7* expression during mandibular osteogenesis.

### 3.4. Identification of HSP47 as a Downstream Target of SP7 and Its Expression During Osteogenic Differentiation in BMSCs with Low SELS Expression

**Figure 4:**
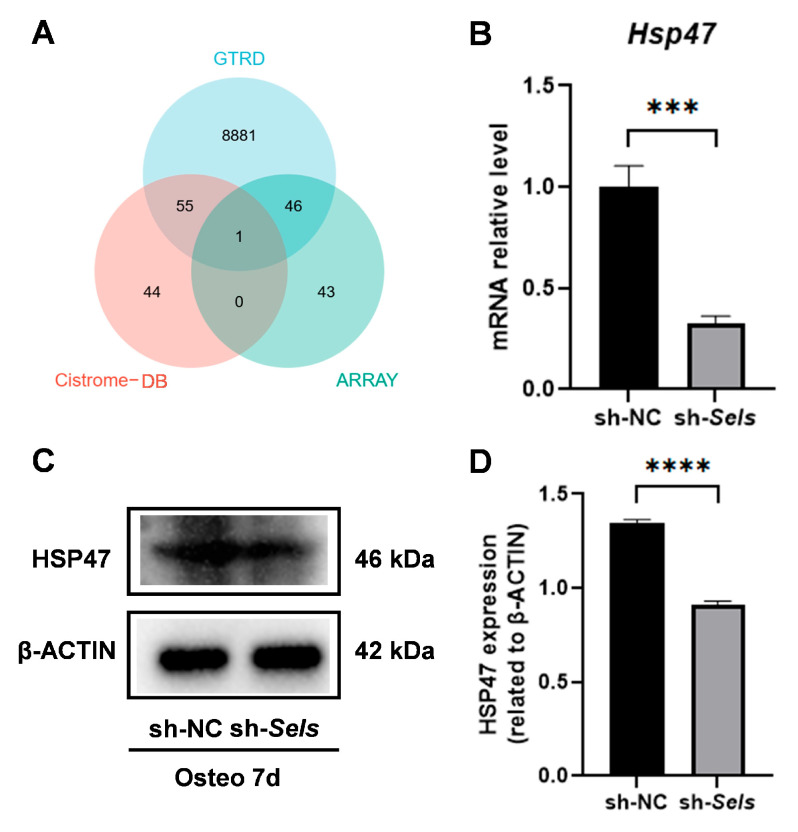
The identification of *HSP47* as a downstream target of *SP7* and its expression during osteogenic differentiation in BMSCs with low SELS expression. (**A**) The potential target gene *HSP47* of *SP7* was identified via combining predictions from the GTRD and Cistrome-GO databases with screening from the osteogenesis-related gene set. (**B**) mRNA expression levels of *HSP47* during osteogenic induction. (**C**,**D**) Changes in *HSP47* protein levels during osteogenic differentiation in cells with low SELS expression. “sh-NC” and “sh-*Sels*” represent the control group and the SelS knockdown group, respectively. Data are represented as means ±SD. (*** *p* < 0.001, **** *p* < 0.0001).

We combined predictions from online databases with an osteogenesis-related gene set and identified *HSP47* as a potential downstream target of *SP7* (Figure 4A). To investigate *HSP47* expression during osteogenic differentiation under SELS knockdown, BMSCs were transduced with lentivirus to silence SELS, achieving approximately 90% reduction in its expression, as confirmed by Western blotting (Supplementary Figure S1). The cells then underwent osteogenic induction. Quantitative PCR analysis revealed a significant decrease in *HSP47* mRNA levels during osteogenic differentiation in SELS-knockdown BMSCs (Figure 4B). Western blot analysis showed a significant decrease in *HSP47* protein levels (Figure 4C,D).

### 3.5. HSP47 Expression Is Regulated by SP7 Binding at Its Promoter Region

**Figure 5:**
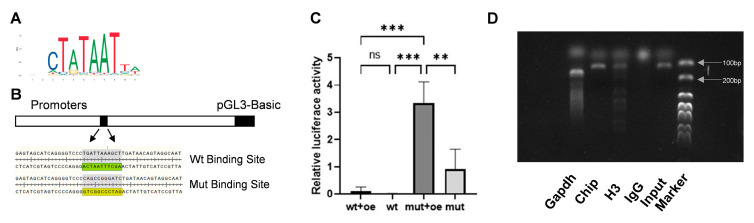
*SP7* can bind to the promoter region of *HSP47* to regulate its expression. (**A**,**B**) Predicted *SP7*-binding sites on the *HSP47* promoter sequence using the JASPAR database, with the predicted and mutated binding sites shown. (**C**) Relative fluorescence expression in luciferase reporter gene assays. (**D**) DNA fragments containing the binding site sequence were successfully amplified in ChIP assays. oe: *SP7* overexpression plasmids; wt: plasmids containing the wild-type promoter sequence, mut: plasmids containing a mutant *SP7*-binding site sequence, re: Renilla luciferase plasmid. Data are represented as means ± SD. (** *p* < 0.01, *** *p* < 0.001, ns, not significant).

To determine whether *SP7* and *HSP47* are transcription factors and target molecules of each other, the JASPAR database was utilized to predict the candidate binding sites and a dual-luciferase reporter assay was conducted. Five binding sites were identified (Table 2), the most probable bases at the binding site are shown, with larger font size indicating a higher likelihood of binding (Figure 5A) and the binding sites were mutated according to the principle of A-C, G-T (Figure 5B). The result of dual-luciferase reporter gene assay demonstrated fluorescence expression after the binding sites were mutated, indicating a potential reverse regulatory relationship between *SP7* and *HSP47* (Figure 5B). To confirm a direct binding relationship between *SP7* and the *HSP47* promoter sequence, we conducted ChIP and found that the *HSP47* promoter sequence containing the *SP7*-binding site could be amplified, indicating a direct binding relationship between *SP7* and the promoter sequence of *HSP47* (Figure 5C).

### 3.6. Upregulation of SP7 Downstream Targets COL1A1 and SPARC Under Conditions of Low SELS Expression

**Figure 6:**
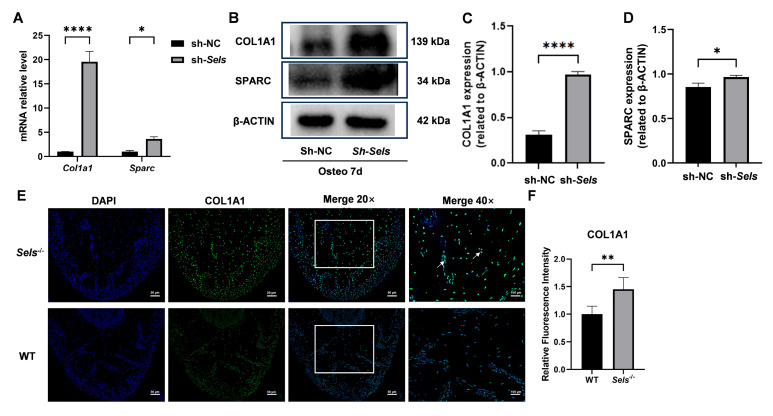
Expression of *SP7* downstream targets *COL1A1* and *SPARC* during osteogenic differentiation in BMSCs with low SELS expression. (**A**) mRNA expression levels of *COL1A1* and *SPARC* after 7 days of osteogenic induction in BMSCs with low SELS expression. (**B**) Protein expression levels of *COL1A1* and *SPARC* in BMSCs with low SELS expression after 7 days of osteogenic induction. (**C**,**D**) Quantification of *COL1A1* and *SPARC* protein levels using ImageJ; values were normalized to β-ACTIN. (**E**) Immunofluorescence staining of *COL1A1* in the furcation region of the mandibular bone from *Sels* knockout (experimental) and wild-type (control) mice. Images within the boxed areas show low-magnification views, with corresponding high-magnification images displayed at the indicated locations. Typical positive cells are marked with arrows. (**F**) Quantitative analysis of relative *COL1A1* fluorescence intensity. “sh-NC” and “sh-*Sels*” represent control and Sels-knockdown BMSCs; “WT” and “*Sels*^−/−^” represent wild-type and Sels knockout mice. Data are represented as means ± SD (*n* = 5), scale bars: 50 and 100 μm. (* *p* < 0.05, ** *p* < 0.01, **** *p* < 0.0001).

To further clarify the downstream mechanisms by which *SP7* mediates the effects of SELS on osteogenesis, we assessed the expression of two known *SP7* target genes: *COL1A1* and *SPARC*. After 7 days of osteogenic induction, both *COL1A1* and *SPARC* showed significantly increased expression at the mRNA and protein levels in BMSCs with low SELS expression compared with controls (Figure 6A–D). Furthermore, immunofluorescence staining demonstrated that *COL1A1* expression was significantly elevated in the mandibular bone of 7-week-old *Sels* knockout mice (experimental group) compared with wild-type mice (control group) (Figure 6E,F).

### 3.7. Mechanism Diagram

**Figure 7:**
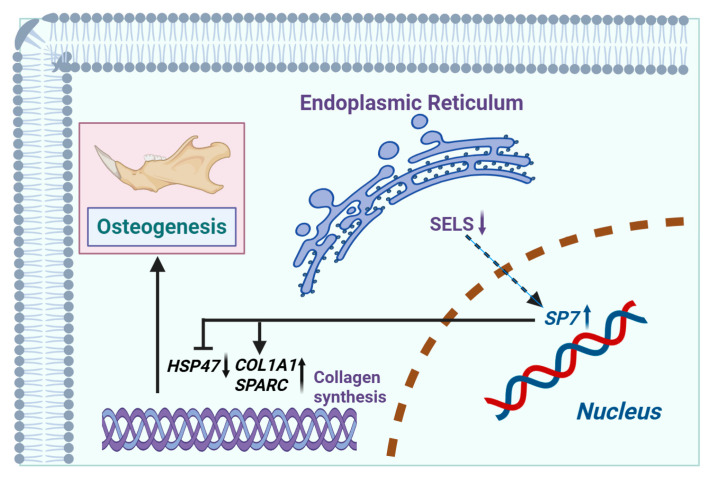
Mechanism via which SELS regulates osteogenic differentiation. SELS, located in the endoplasmic reticulum, indirectly modulates the expression of the osteogenesis-related transcription factor *SP7* (with *SP7* expression increasing as SELS expression decreases). *SP7* binds to the promoter regions of downstream targets, regulating the expression of *HSP47*, *COL1A1*, and *SPARC* (negatively regulating *HSP47* and positively regulating *COL1A1* and *SPARC*). This regulation affects collagen synthesis, thereby influencing osteogenic differentiation.

The mechanism via which SELS regulates osteogenic differentiation through *SP7* by modulating the expression of *HSP47*, *COL1A1*, and *SPARC* is shown in Figure 7. SELS downregulation leads to increased expression of *SP7*, which subsequently regulates the expression of *HSP47*, *COL1A1*, and *SPARC*. By upregulating *COL1A1* and *SPARC* and downregulating *HSP47*, *SP7* mediates the regulation of osteogenic differentiation.

## 4. Discussion

In this study, we integrated bioinformatics with in vivo and in vitro experiments to assess the role of SELS in osteogenic differentiation. SELS expression was elevated in the mandibles of 3-week-old wild-type mice, representing the active ossification stage, compared to that in 12-week-old wild-type mice, which represent the stage of stable osteogenic differentiation. Through in vitro osteogenic induction of BMSCs with SELS knockdown, and assessment of *SP7* expression in the mandibles of 7-week-old *Sels* knockout mice, we found that low SELS expression led to upregulation of the osteogenic transcription factor *SP7*. Transcriptomic analysis suggested that SELS may regulate osteogenesis by modulating collagen synthesis and extracellular matrix mineralization. We identified *HSP47* as a downstream target of *SP7*, with luciferase and ChIP assays confirming its negative regulation. In BMSCs with low SELS expression, *HSP47* was downregulated, while the *SP7* targets *COL1A1* and *SPARC* were upregulated. These findings support the idea that the SELS–*SP7*–*HSP47*/*COL1A1*/*SPARC* axis plays a role in regulating osteogenesis (Figure 7).

Our earlier research revealed that the expression of SELS was altered during osteogenic differentiation, with discordant expression trends between mRNA and protein [14]. Research has demonstrated that dexamethasone can induce the degradation of SELS [18]; therefore, we propose that this inconsistency may be attributed to the presence of dexamethasone in the osteogenic induction medium. To avoid such interference, we conducted in vivo experiments and found that SELS protein levels were elevated in the mandibular bone of 3-week-old mice, a stage associated with active osteogenesis. This finding indicates that SELS expression is elevated during the active stage of osteogenic differentiation, while SELS downregulation can promote osteogenic differentiation. Although this result may appear contradictory and potentially confusing to readers, it is not necessarily conflicting. Similar functional phenomena have been reported for molecules such as Sclerostin (SOST) [19].

To explore the regulatory role of SELS in osteogenic differentiation, we first assessed its expression dynamics and reanalyzed transcriptomic data previously generated and published by our research group [14]. In the mandibular dataset, DEGs associated with SELS were enriched in collagen synthesis, extracellular matrix (ECM) organization, and matrix mineralization. Similar results were observed in BMSCs with low SELS expression, highlighting SELS-related involvement in collagen production [20], ECM formation, and mineralization [21]. These results, together with previous findings linking selenoproteins to collagen biosynthesis [22,23], suggest that SELS may regulate osteogenic differentiation primarily by modulating collagen synthesis and matrix mineralization. During bone formation, mesenchymal stem cells differentiate into osteoblasts that synthesize and secrete type I collagen fibers—the main organic component of the bone matrix and a critical scaffold for subsequent mineral deposition. Proper collagen production is, therefore, fundamental to bone formation [24]. Based on these findings, we prioritized he investigating osteogenesis-related markers involved in collagen production and extracellular matrix organization to further elucidate the regulatory role of SELS.

The roles of selenium and its associated selenoproteins in the human body are primarily related to antioxidation and homeostasis [25,26]. Several studies have shown that the dysregulation of selenoproteins such as GPX4 and GPX7 affects osteogenic differentiation, largely through modulating intracellular oxidative stress [27,28]. SELS is one of the 25 selenoproteins identified in the human body. It is mainly distributed within the endoplasmic reticulum and around the nucleus [2]. While SELS has been associated with ER stress, oxidative stress, and inflammatory responses, emerging evidence suggests that it may also regulate cytokine and transcription factor activity [4,29]. Since stable expression of transcription factors and cytokines is essential for normal osteogenesis [4], we investigated the role of SELS in this process. Our results showed that low SELS expression led to the upregulation of *SP7*, an osteoblast-specific transcription factor widely recognized as a key regulator and marker of osteogenic differentiation [30,31]. *SP7* activates several downstream genes, including *COL1A1*, *SPARC*, and OCN, whose functions are associated with collagen synthesis and cell mineralization. Increased *SP7* expression has been shown to promote the expression of these molecules, thereby enhancing osteogenesis [8]. However, osteogenesis must be tightly controlled to avoid pathological bone formation. While *SP7* is well known for its role in activating pro-osteogenic genes, whether it also contributes to negative regulatory feedback remained unclear. Interestingly, previous studies reported that Fibrillin-2 and Periostin levels increase when *SP7* expression is reduced [13], hinting at a potential bidirectional regulatory mechanism. These findings highlight the importance of balanced positive and negative regulation during osteogenesis, though the exact nature of *SP7*’s interactions with its downstream targets requires further investigation.

To address this gap, we identified *HSP47* as a candidate downstream gene of *SP7*. We observed that *HSP47* expression was significantly reduced during osteogenic induction in BMSCs with low SELS expression, which also exhibited increased *SP7* levels. Dual-luciferase reporter assays and ChIP experiments confirmed that *SP7* directly interacts with the *HSP47* promoter sequence and negatively regulates its transcription. These findings indicate that *HSP47* may act as a negative regulator in osteogenesis, helping to prevent excessive bone formation. *HSP47*, a member of the serpin protein family, acts as an essential chaperone in the endoplasmic reticulum [32], ensuring the proper folding of collagen [33]. The absence of *HSP47* results in improper collagen folding. Researchers have declared that the function of *HSP47* is closely related to osteogenesis, with its loss in vivo potentially causing osteogenesis imperfecta type 10 [34]. Furthermore, the abnormal expression of *HSP47* could lead to the manifestation of various diseases [35]. *HSP47* binds to type I collagen to prevent its premature folding or aggregation, thereby ensuring the proper formation of its tertiary structure [36]. During matrix mineralization, *HSP47* facilitates the correct arrangement and mineralization of collagen fibers [37]. Notably, *HSP47* functions as a partner molecule of *COL1A1*, with both serving as downstream target molecules of *SP7*.

*COL1A1* and *SPARC* are well-established *SP7* target genes and classical markers of osteogenic differentiation. In this study, their expression was significantly upregulated during osteogenic induction in BMSCs with low SELS expression, supporting their roles as positive effectors of *SP7*-mediated osteogenesis. *COL1A1* is the main component of type I collagen and is essential for its proper assembly [38]. *SPARC*, also known as osteonectin, is a matricellular protein, participates in extracellular matrix formation, and interacts with multiple types of collagens to regulate synthesis and mineralization [39]. Its two calcium-binding domains enable interaction with calcium ions, further modulating mineralization. Interestingly, in contrast to *COL1A1* and *SPARC*, which are positively regulated by *SP7*, *HSP47* is negatively regulated. This opposing pattern suggests that *SP7* coordinates both positive and negative regulatory pathways to fine-tune osteogenic differentiation. While *COL1A1* and *SPARC* promote matrix formation and mineralization, *HSP47* may function as a brake to prevent excessive ossification. This balancing mechanism is likely critical for maintaining tissue integrity and preventing pathological bone formation. Based on these findings, we propose that low expression of SELS modulates osteogenesis via *SP7* by simultaneously upregulating *COL1A1* and *SPARC* while downregulating *HSP47*, thereby exerting precise bidirectional control over osteogenic differentiation. Such fine-tuned regulation ensures coordinated matrix deposition and mineralization, preventing uncontrolled bone formation that could lead to pathological conditions. Moreover, elucidating this SELS–*SP7*–*HSP47*/*COL1A1*/*SPARC* axis provides valuable insights into the transcriptional networks governing bone development and offers potential molecular targets for therapeutic strategies aimed at promoting bone regeneration or treating bone loss and mandibular defects.

## 5. Conclusions

Our study demonstrates that SELS expression increases during the active phase of osteogenic differentiation. Low SELS expression promotes osteogenic differentiation by upregulating *SP7*, which in turn suppresses *HSP47* while activating its downstream targets *COL1A1* and *SPARC*, thereby enabling precise regulation of bone formation. These findings elucidate a novel *SP7*–*HSP47*/*COL1A1*/*SPARC* regulatory axis in osteogenesis and provide potential molecular targets and a theoretical basis for the prevention and treatment of bone loss and defects.

## Figures and Tables

**Table 1:** Primers for qPCR.

Genes	Forward	Reverse
*Gapdh*	5′-TTGATGGCAACAATCTCCAC-3′	5′-CGTCCCGTAGACAAAATGGT-3′
*Selenos*	5′-GCТGGСТАGТТGGТАGGТТGА-3′	5′-CGCATCCCGTCACAGAGA-3′
*HSP47*	5′-GCAGCAGCAAGCAACACTACAACT-3′	5′-AGAACATGGCGTTCACAAGCAGTG-3′
*COL1A1*	5′-GCTCCTCTTAGGGGCCACT-3′	5′-CCACGTCTCACCATTGGGG-3′
*SPARC*	5′-ACCCCCGGCAATTTCATGG-3′	5′-TGTCTTCCCAGCTCTTGATGTAA-3′

**Table 2:** Predicted *SP7*-binding motifs in the *HSP47* promoter.

Matrix ID	Name	Score	Sequence ID	Start	End	Strand	Predicted Sequence
UN0264.1	UN0264.1.*SP7*	8.447574	NC_000073.7:c99004321-99002222	1515	1525	−	AGCTTTAATCA
UN0264.1	UN0264.1.*SP7*	8.249999	NC_000073.7:c99004321-99002222	22	32	−	TTCTATTATCA
UN0264.1	UN0264.1.*SP7*	7.20972	NC_000073.7:c99004321-99002222	1379	1389	+	AGCTAGAATTC
UN0264.1	UN0264.1.*SP7*	7.020698	NC_000073.7:c99004321-99002222	1264	1274	+	TGCTGTATTTA
UN0264.1	UN0264.1.*SP7*	6.8002434	NC_000073.7:c99004321-99002222	615	625	+	CTCTATAACTG

## Data Availability

The raw data supporting the conclusions of this article will be made available by the authors on request.

## Supplementary Materials

The following supporting information can be downloaded at https://www.mdpi.com/article/10.3390/cimb47090677/s1.
