# Supplementary material for: Low Expression of Selenoprotein S Modulates Osteogenic Differentiation Through Bidirectional Regulation of the SP7–HSP47/COL1A1/SPARC Axis
Source: Curr Issues Mol Biol. 2025 Aug 23;47(9):677. doi: 10.3390/cimb47090677 (PMC12468540; doi:10.3390/cimb47090677)
Supplement: Supplementary file 1 [file cimb-47-00677-s001.zip › cimb-3781776-supplementary.pdf]

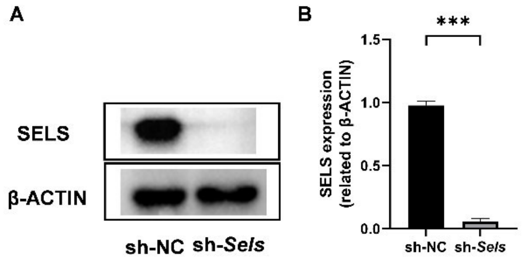

Figure S1 Validation of SELS knockdown efficiency in BMSCs. (A) Western blot analysis showing the protein levels of SELS in BMSCs transduced with control or SELS-targeting lentivirus. (B) Quantitative analysis of SELS protein levels normalized to β-ACTIN.
